# Sonographic Corroborative Dissection of C5 –T1 Ventral Rami of Brachial Plexus© with Sonographic Signs for Identification

**DOI:** 10.5152/TJAR.2022.21291

**Published:** 2022-12-01

**Authors:** Archana Areti, Sivashanmugam T, Jaya Velraj, Prabhavathy Gajendran

**Affiliations:** 1 Department of Anaesthesia, Mahatma Gandhi Medical College & Research Institute, Sri Balaji Vidyapeeth Deemed-to-be University, Puducherry, India; 2 Department of Anaesthesia, JIPMER, Karaikal, Puducherry, India

High definition ultrasonography (US) of the 5 ventral rami (VR) of brachial plexus (BP) has been demonstrated;^1^ however, identification of C8-T1VR was most challenging.^2^ This illustration provides cadaveric images corresponding to the sonoanatomy of C5–T1VR and also sonographic signs for easy identification (Figure 1). The images were obtained by sonographic corroborative dissection method^©^.^3^ The yellow arrows indicate the VR, green arrows indicate bony reference point for level identification. C5, C6, C7, & C8VR were assigned sunrise signs (static signs), as the hypoechoic VR could be likened to a sun rising between 2 mountains for C5 and C6 (A-B), 1 mountain for C7 (C), and the sea for C8 (D). The origin of T1VR was not visible on US as it originates and courses below the proximal part of the first rib.^2^ It can be visualized as it climbs over the medial border of the first rib to join C8VR forming the inferior trunk where it appears as if it is taking a “U” turn. Hence a dynamic sign has been assigned for T1VR (“U Turn sign”). These US signs not only help in identification of the VR objectively and consistently but also facilitate effective teaching and training of brachial plexus imaging. 

## Figures and Tables

**Figure 1.**
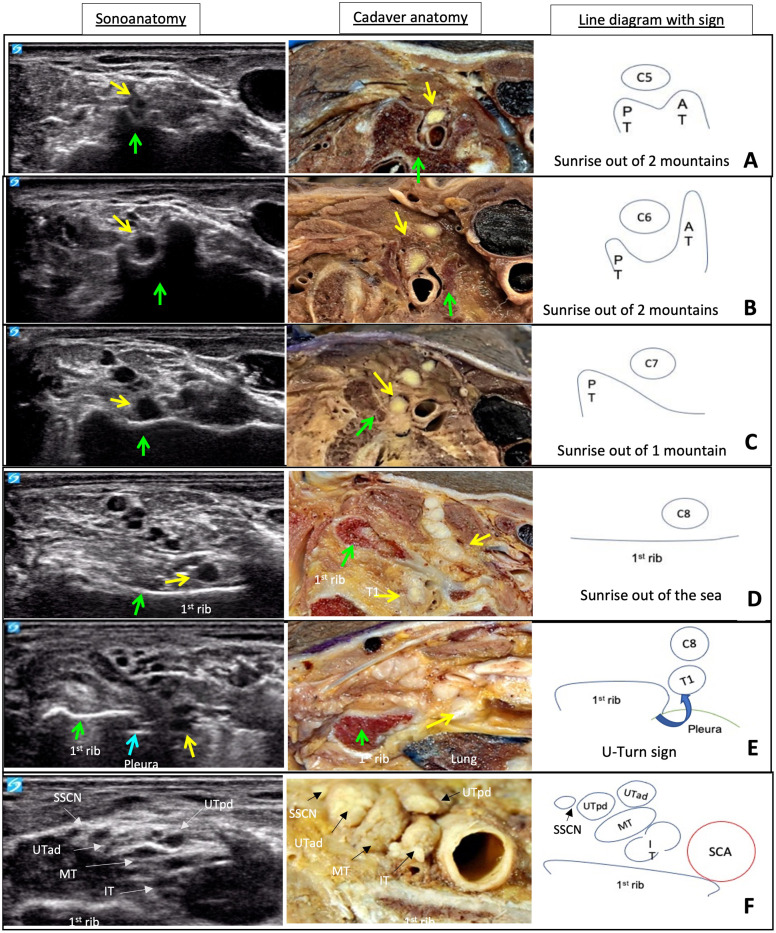
Image showing the sonographic corroborative cadaveric sections and line diagram representation of the sonoanatomy at the different levels of the brachial plexus. SCA, subclavian artery; SSCN, suprascapular nerve; UTpd, upper trunk posterior division; UTad, upper trunk anterior division; MT, middle trunk; IT, inferior trunk.
